# Optimized dose regimen for whole-body FDG-PET imaging

**DOI:** 10.1186/2191-219X-3-63

**Published:** 2013-08-12

**Authors:** Eleonore H de Groot, Nieky Post, Ronald Boellaard, Nils RL Wagenaar, Antoon TM Willemsen, Jorn A van Dalen

**Affiliations:** 1 Department of Nuclear Medicine and Molecular Imaging, University of Groningen, University Medical Center Groningen, P.O. Box 30.001, Groningen 9700 RB, The Netherlands; 2 Department of Nuclear Medicine, Ziekenhuisgroep Twente, P.O. Box 546, Hengelo 7550 AM, The Netherlands; 3 Department of Radiology and Nuclear Medicine, VU University Medical Centre, de Boelelaan 1117, Amsterdam 1081 HV, The Netherlands; 4 Department of Medical Physics, Isala Klinieken, Groot Wezenland 20, Zwolle 8011 JW, The Netherlands

**Keywords:** Positron-emission tomography, Image quality, Weight dependency, Signal-to-noise ratio, Dose

## Abstract

**Background:**

The European Association of Nuclear Medicine procedure guidelines for whole-body fluorodeoxyglucose positron-emission tomography (FDG-PET) scanning prescribe a dose proportional to the patient’s body mass. However, clinical practice shows degraded image quality in obese patients indicating that using an FDG dose proportional to body mass does not overcome size-related degradation of the image quality. The aim of this study was to optimize the administered FDG dose as a function of the patient’s body mass or a different patient-dependent parameter, providing whole-body FDG-PET images of a more constant quality.

**Methods:**

Using a linear relation between administered dose and body mass, FDG-PET imaging was performed on two PET/computed tomography scanners (Biograph TruePoint and Biograph mCT, Siemens). Image quality was assessed by the signal-to-noise ratio (SNR) in the liver in 102 patients with a body mass of 46 to 130 kg. Moreover, the best correlating patient-dependent parameter was derived, and an optimized FDG dose regimen was determined. This optimized dose regimen was validated on the Biograph TruePoint system in 42 new patients. Furthermore, this relation was verified by a simulation study, in which patients with different body masses were simulated with cylindrical phantoms.

**Results:**

As expected, both PET systems showed a significant decrease in SNR with increasing patient’s body mass when using a linear dosage. When image quality was fitted to the patient-dependent parameters, the fit with the patient’s body mass had the highest *R*^2^. The optimized dose regimen was found to be *A*_new_*= c*/*t × m*^2^, where *m* is the body mass, *t* is the acquisition time per bed position and *c* is a constant (depending on scanner type). Using this relation, SNR no longer varied with the patient’s body mass. This quadratic relation between dose and body mass was confirmed by the simulation study.

**Conclusion:**

A quadratic relation between FDG dose and the patient’s body mass is recommended. Both simulations and clinical observations confirm that image quality remains constant across patients when this quadratic dose regimen is used.

## Background

The European Association of Nuclear Medicine (EANM) procedure guidelines for whole-body fluorodeoxyglucose positron-emission tomography(/computed tomography) (FDG-PET(/CT)) scans for tumour imaging [1] provide a standardization for the administration of FDG and the acquisition and reconstruction of an FDG whole-body PET scan. Together with quality control standards, these guidelines ensure that the measured FDG tumour uptake is, within certain limits, independent of the system used or the centre where the study is performed. Furthermore, it was discussed that the linear relationship between the patient’s body mass and administered FDG dose would result in a more uniform image quality between patients compared to a constant administered dose. However, it is well known from clinical practice that even after adherence to the guidelines, the image quality of whole-body FDG-PET scans decreases for obese patients, which can result in false-negative PET scans. Thus, a different relationship between dose and body mass or a different patient-dependent parameter, e.g. body mass index (BMI) or lean mass, might be required to obtain an even more constant image quality [2,3]. Research on image quality has been performed at various institutes [4,5], also with a special focus on obese patients [6,7]. Using the signal-to-noise ratio (SNR) in the liver as a measure for image quality, the use of a higher FDG dose per kilogram of body mass (typically 6 to 10 MBq/kg) or longer PET acquisition times per bed position for patients with high body mass were suggested [5,8]. However, the optimal relationship has not yet been determined. For children, an optimized dose regimen using an exponential relation between body mass and FDG dose was suggested [9].

Thus, the aim of this study was to optimize the administered FDG dose as a function of a patient-dependent parameter, providing whole-body FDG-PET images of a more constant quality.

Since the publication of [1], more PET cameras have been equipped with a time of flight (TOF) option [10-12]. In addition, reconstructions using a position-dependent point spread function (PSF) have also been introduced [13]. It is possible that these new reconstruction options change the optimal relation between the patient-dependent parameter and the administered FDG dose. Therefore, these different reconstruction methods were taken into account in this study.

## Methods

For all parts of the study, the patients were scanned according to the standard clinical protocol valid at the moment of their scan at their hospital. Based on the outcome of the first part of the study, the standard clinical protocol at the site of the Biograph TruePoint was changed. So, in this study all analyses were performed retrospectively on anonymized clinical patient data. Therefore, approval by the medical ethics committee was not required.

The statistical analysis was performed using SigmaPlot for Windows, version 10.0 (Systat Software Inc., Chicago, IL, USA).

### First part of the study

First, a retrospective analysis was performed on the image quality of whole-body FDG-PET scans for two groups of patients in two hospitals. For both groups, local administration and scanning protocols were followed. One group, consisting of 40 patients, underwent PET/CT on a Siemens Biograph 40 TruePoint camera with TrueV (Knoxville, TN, USA). The second group, consisting of 62 patients, underwent PET/CT on a Siemens Biograph mCT camera with a 64-slice CT. An overview of patient characteristics and acquisition parameters is given in Table 1. In all cases, PET images were reconstructed using three-dimensional ordered subset expectation maximization (OSEM3D), with CT-based attenuation correction. For the Biograph mCT, also the position-dependent PSF and TOF options were used. The reconstruction parameters that were used are summarized in Table 2. The patients for both the first and the second part of the study were primarily selected consecutively from the patients that were scanned in the hospitals. However, heavier patients (>90 kg) were specifically selected in the end to obtain a better spread in the patient’s body mass. On the Biograph mCT, all patients were scanned with their arms above their head; on the Biograph TruePoint, part of the patient group was scanned with their arms along their body.

**Table 1:** Patient characteristics and acquisition parameters

	**Part of study**		
	**First part**	**First part**	**Second part**
Camera	Biograph TruePoint	Biograph mCT	Biograph TruePoint
Number of patients	40	62	42
Body mass (kg)			
Mean ± SD	80.9 ± 21.1	82.6 ± 18.0	80.3 ± 23.7
Range	50 to 130	46 to 125	45 to 125
Length (m)			
Mean ± SD	1.72 ± 0.10	1.76 ± 0.11	1.73 ± 0.10
Range	1.52 to 1.95	1.53 to 1.99	1.45 to 1.99
Time between administration and scan (min), (range)	60 (53 to 65)	60 (55 to 65)	63 (54 to 98)
PET acquisition mode	3D	3D	3D
Prescribed FDG dose (MBq)			
Body mass < 90 kg	2.13·*m*	5·*m*	0.023·(*m*^2.047^)
Body mass > 90 kg	191.7 + 5·(*m* − 90)	5·*m*	0.023·(*m*^2.047^)
Acquisition time per bed position (min)			
Body mass < 60 kg	4	1	4
Body mass = 60 to 90 kg	4	2	4
Body mass > 90 kg	4	3	4

**Table 2:** Reconstruction parameters used on the two PET/CT cameras

**Camera**	**Reconstruction**	**Iterations**	**Subsets**	**Gaussian filter (mm)**
Biograph TruePoint	OSEM3D	4	8	4
Biograph mCT	OSEM3D	3	24	5
Biograph mCT	OSEM3D + PSF	3	24	5
Biograph mCT	OSEM3D + PSF + TOF	3	21	5

As a measure of image quality, the SNR in the liver was used as it is the only organ in the human body that has a relatively homogeneous uptake of FDG. Patient scans with inhomogeneous uptake, particularly due to liver metastasis or other irregularities in the liver, were excluded from this study. The SNR is defined as the ratio of the mean pixel value (mean) to the standard deviation (SD) in the observed region:

(1) $$\mathrm{SNR}=\frac{\mathrm{Mean}}{\mathrm{SD}}.$$

As nuclear positron emission is a Poisson-distributed random process, PET is a statistical imaging technique where in the first-order approximation we expect that [4]:

(2) $$\mathrm{SNR}=\frac{\mathrm{Mean}}{\mathrm{SD}}\sim \frac{N}{\sqrt{N}}=\sqrt{N}\sim \sqrt{A\cdot t},$$

where *N* is the number of disintegrations measured, *A* is the activity (MBq) in the region of interest measured and *t* is the scan time per bed position (min).

The SNR in the liver (SNR_L_) was calculated as follows. The transverse CT slice in which the liver has the largest cross section was determined. In the corresponding PET slice and three adjacent slices, a region of interest (ROI) was drawn in the liver, in which the mean pixel value and standard deviation were measured. The ROI was identical in all four slices and was drawn far enough from the edges of the liver to avoid partial volume effects. The SNR_L_ was calculated for each ROI using Equation 1 and was then averaged for the four ROIs. To investigate whether SNR_L_ decreases significantly with body mass, it was corrected for the different scan times per bed position, by dividing it by the square root of the acquisition time per bed position (based on Equation 2). Then a linear regression of SNR_L_/√*t* with body mass was performed. The slope of the regression line was regarded to be significantly different from zero if *p* < 0.05.

Using Equation 2, an SNR_L_ normalized for the administered dose and scan time per bed position was defined as given in Equation 3 (SNR_norm_ in (MBq min)^−1/2^):

(3) $${\mathrm{SNR}}_{\mathrm{norm}}=\frac{{\mathrm{SNR}}_{L}}{\sqrt{A\cdot t}},$$

where *A* is the administered dose (MBq) and *t* is the acquisition time per bed position (min).

The uptake of FDG is related to body mass, but alternative patient-dependent parameters may be more appropriate [2]. Therefore, SNR_norm_ was displayed as a function of various patient-dependent parameters and fitted with a single polynomial function, given by Equation 4. The 95% confidence intervals (95% CIs) were also calculated for these fits:

(4) $${\mathrm{SNR}}_{\mathrm{fit}}=a\cdot p^{-d},$$

where SNR_fit_ ((MBq min)^−1/2^) is the result of the fit, *a* and *d* are fit parameters and *p* is a patient-dependent parameter.

The tested patient-dependent parameters were body mass, BMI, lean body mass as defined by Hume [14] and James [15], fat mass (defined by body mass minus the lean body mass) and body mass per body length. These parameters were chosen because they are related to body shape and fat content. Based on the coefficient of determination (*R*^2^), the best patient-dependent parameter was selected. The relative error between SNR_norm_ and SNR_fit_ was calculated for each data point using (SNR_fit_ − SNR_norm_)/SNR_fit_ × 100%. An *F* test was performed to test if the standard deviation of the relative error distribution of the different fits differed significantly from that of the fit with the patient-dependent parameter with the highest *R*^2^. The level of statistical significance was set to 0.05 (not corrected for multiple comparisons). Combination of Equations 3 and 4 shows that SNR_L_, and hence the image quality, is constant if

(5) $$\sqrt{A\cdot t}\cdot a\cdot p^{-d}=\mathrm{constant}.$$

The value of the constant in Equation 5 equals an acceptable SNR_L_ (SNR_acc_), which is the value of the SNR_L_ corresponding to the highest body mass for which the image quality is still acceptable. This SNR_acc_ was chosen by an experienced nuclear medicine physician. In this study this was done for the Biograph TruePoint. From Equation 5, it follows that the dose regimen described by Equation 6 should result in a more constant image quality for FDG whole-body PET scans, independent of the patient-dependent parameter:

(6) $$A\cdot t={\left(\frac{{\mathrm{SNR}}_{\mathrm{acc}}}{a\cdot p^{-d}}\right)}^{2}={\left(\frac{{\mathrm{SNR}}_{\mathrm{acc}}}{a}\right)}^{2}\cdot p^{2d}.$$

### Second part of the study

After determination of the optimized dose regimen for the Biograph TruePoint PET/CT device, it was implemented as the new routine clinical protocol. From the ensuing pool of clinical patient data, 42 scans were extracted and analyzed to investigate the effect of this new clinical protocol on image quality. An overview of patient characteristics and acquisition parameters is given in Table 1. The analysis of the SNR_L_ was repeated for these 42 patients, and it was checked using linear regression if the SNR_L_ was independent of the patient’s body mass for this group. The slope of the regression line was regarded to be significantly different from zero if *p* < 0.05.

### Simulation study

In order to support clinical findings and to get a more detailed understanding of the relationship between the administered FDG dose as a function of the patient’s body mass and image quality, several 2D simulations were performed. Patients with different body masses were simulated with a cylindrical mathematical phantom having different diameters of 15, 20, 25 and 30 cm. Assuming a body length of 1.75 m, these diameters correspond to body masses of, respectively, approximately 31, 55, 85 and 125 kg. For each simulated phantom, an emission sinogram was generated by forward projection of the image to sinogram space (144 angles, 4-mm bins, image matrix = 128 × 128). Effects of attenuation were included assuming a uniform water equivalent density in the mathematical phantom (attenuation coefficient *μ* = 0.095 cm^−1^). Poisson noise was then added to the attenuated sinograms, and a total of 50 noisy sinogram replicates were generated. Dead time and contribution of random coincidences and scattered photons were not included. Next, each of the 50 sinograms was reconstructed using attenuation-weighted ordered subset expectation maximization (AW-OSEM) using 4 iterations and 16 subsets. Simulations were performed using three FDG dose-body mass relations: (1) a fixed dose independent of the patient’s body mass, resulting in decreasing activity concentration with increasing patient’s body mass in the simulated image; (2) a linear relation between the patient’s body mass and the administered dose, resulting in a constant activity concentration in the patient (or mathematical phantom), as currently recommended by the EANM guidelines for quantitative FDG PET/CT imaging in oncology [1]; and (3) a quadratic relation between the patient’s body mass and the administered dose, resulting in an increase of activity concentration in the patient (phantom) with increasing body mass. A constant scan duration was assumed for all simulations. To simplify the comparison between the three relations, the simulations are designed such that the 20-cm-diameter phantom data provide equal results for the three different dose regimes. The SNR in the reconstructed images was assessed within a circular ROI with a diameter which was 4 cm less than the diameter of the simulated cylinder (i.e. the 2-cm outer edge of the phantom was not included). The values of the SNR seen in individual images were then averaged over all 50 replicates to obtain a more precise estimate of image SNR.

## Results

### First part of the study

**Figure 1:**
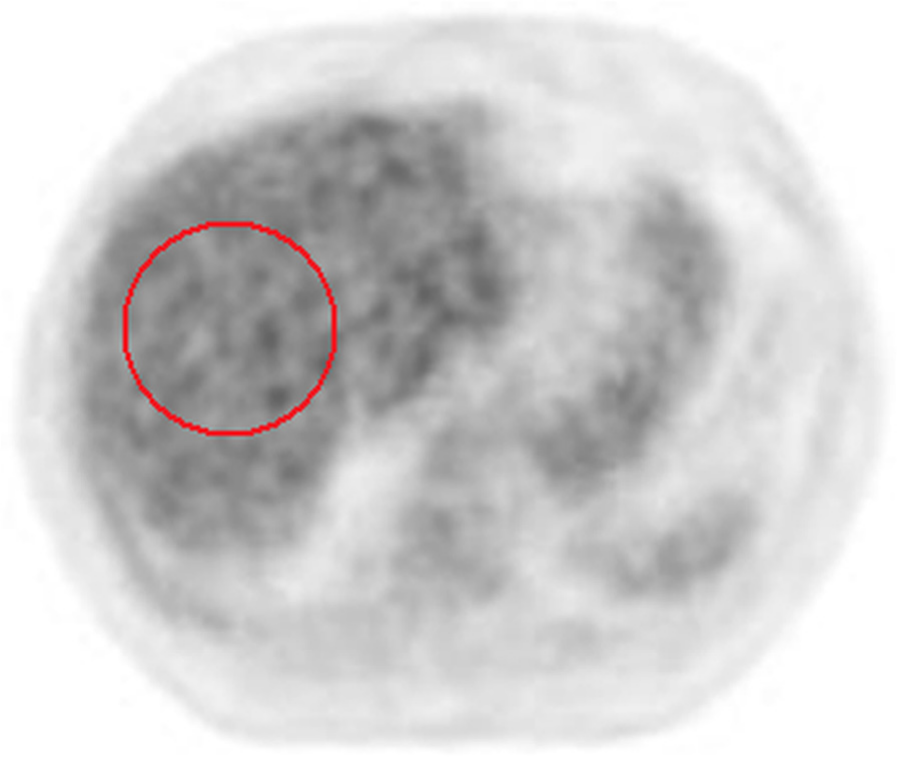
**Axial slice with ROI.** An axial slice of one of the patient scans showing the ROI used to derive the SNR in the liver (red contour).

**Figure 2:**
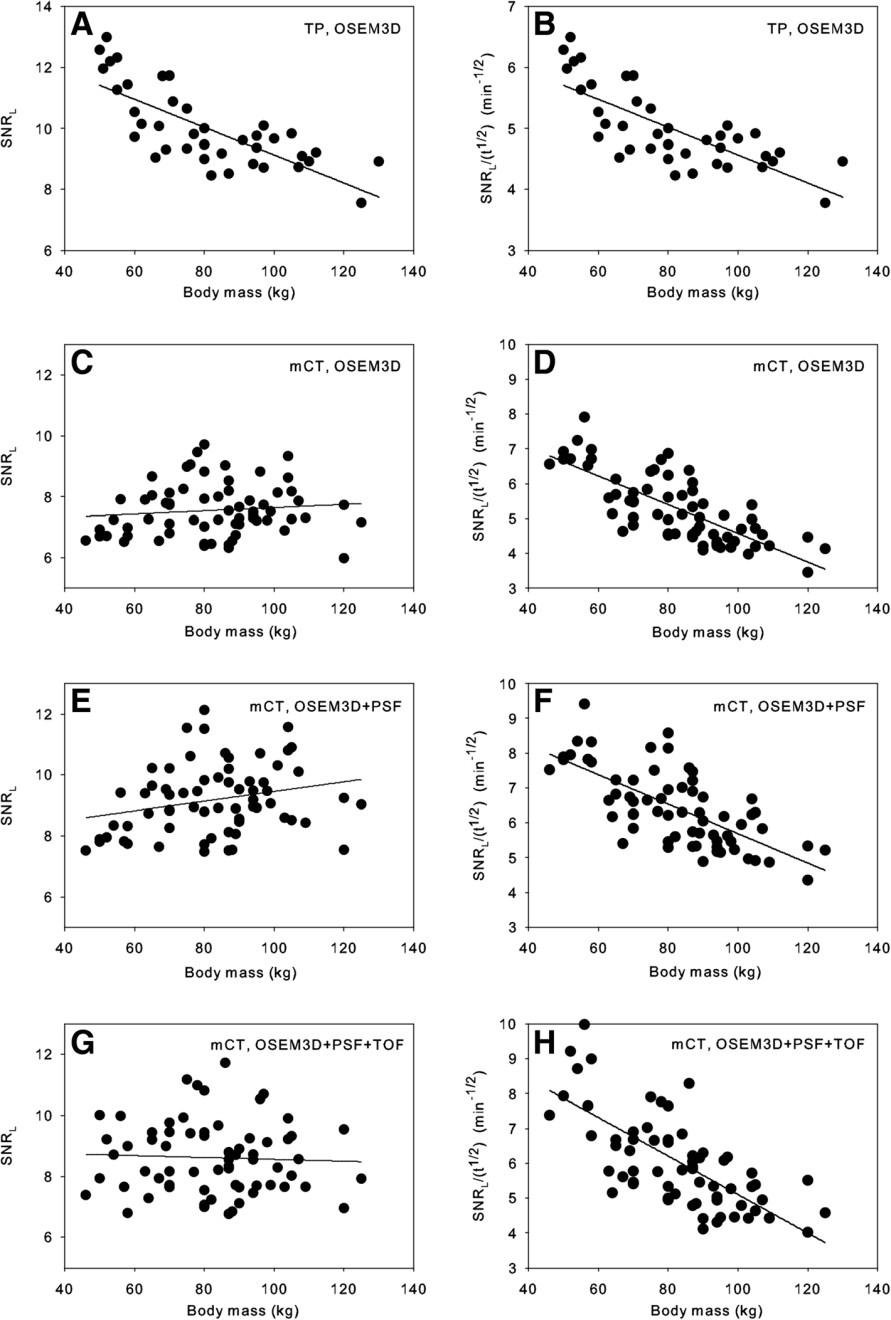
**SNR**_**L **_**and SNR**_**L**_**/(*t***^**1/2**^**) versus body mass.** Signal-to-noise ratio in the liver (SNR_L_) versus body mass (left) and SNR_L_ normalized to an acquisition time per bed position of 1 min versus body mass (right) for the Biograph TruePoint (TP), OSEM3D reconstruction **(A**, **B)** and the Biograph mCT for three different reconstructions: **(C**, **D)** OSEM3D, **(E**, **F)** OSEM3D + PSF, and **(G**, **H)** OSEM3D + PSF + TOF. The lines in the graphs are the result of linear regression of the data.

**Figure 3:**
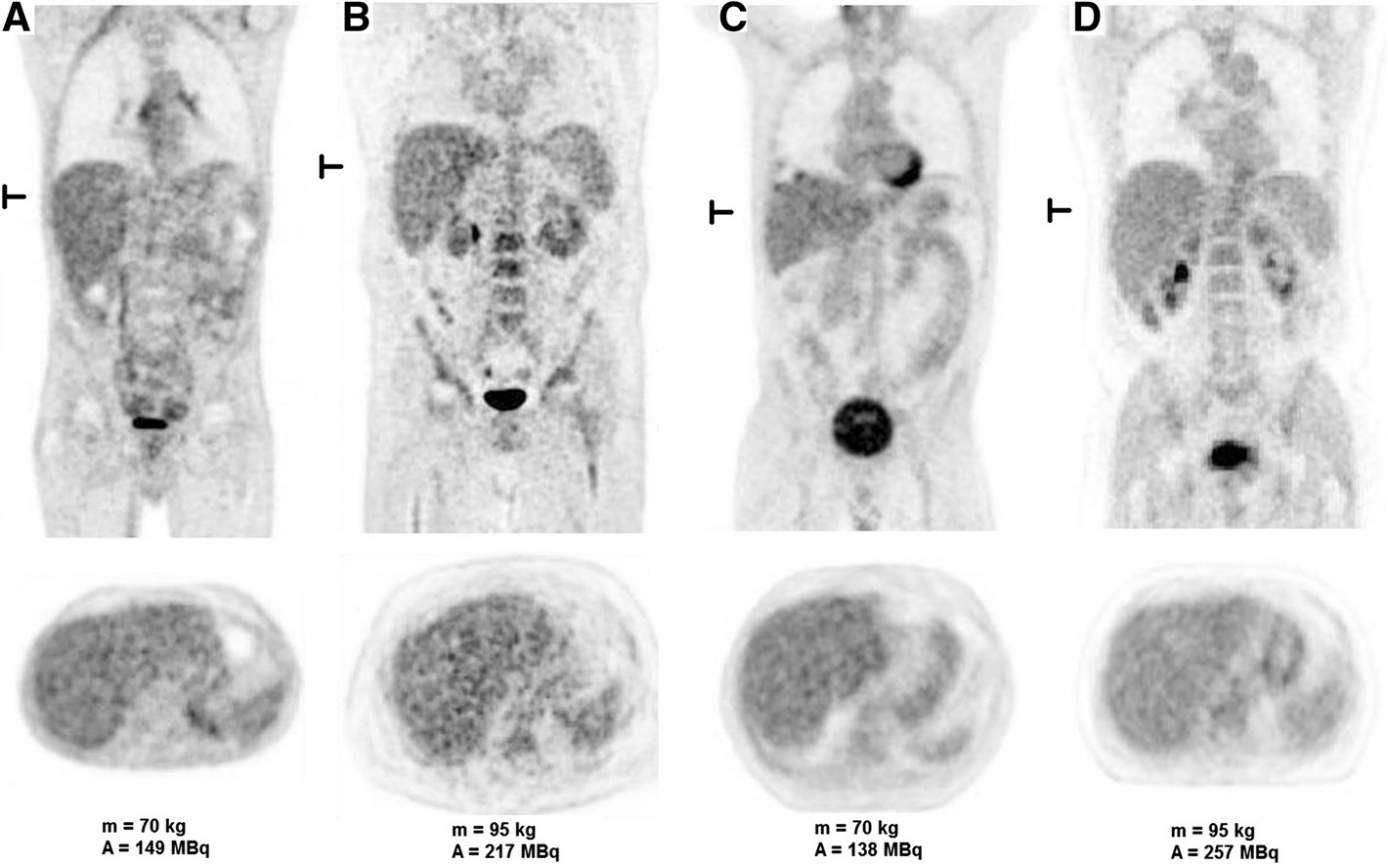
**Patient scans in the old and new dose regimens.** Coronal and transverse slices of a whole-body FDG-PET scan for **(A)** a patient of 70 kg (dose = 149 MBq) and **(B)** a patient of 95 kg (dose = 217 MBq), using the old dose regimen, and for **(C)** a patient of 70 kg (dose = 138 MBq) and **(D)** a patient of 95 kg (dose = 257 MBq), using a quadratic relation between body mass and FDG dose. Using the old relation between body mass and dose, the dose would have been **(C)** 149 and **(D)** 217 MBq, respectively. All four scans were performed on the Biograph TruePoint. The location of the transverse slice is indicated on the coronal view.

Figure 1 shows an example of a ROI used to determine the SNR_L_. Figure 2A shows the relation between the measured SNR_L_ and the patient’s body mass for the Biograph TruePoint after OSEM3D reconstruction. Figure 2B shows the relation between the SNR_L_ corrected for the acquisition time per bed position and the patient’s body mass for the same camera. Figure 2C,E,G shows the relation between the SNR_L_ and the patient’s body mass for the Biograph mCT. Figure 2D,F,H shows the relation between the SNR_L_ corrected for the acquisition time per bed position and the patient’s body mass for the Biograph mCT for all three reconstructions. Figure 2C,E,G shows that the applied relation between body mass and acquisition time per bed position for the Biograph mCT compensates well for the SNR_L_ decline normally seen (*cf.* Figure 2A) in heavy patients. After normalization for the acquisition time per bed position (Figure 2B,D,F,H), a significant decrease in the SNR_L_ with body mass was observed for both cameras (*p* < 0.001). This is illustrated in Figure 3A,B, showing a scan of a patient with a body mass of 70 kg and that of a patient with a body mass of 95 kg, respectively.

**Figure 4:**
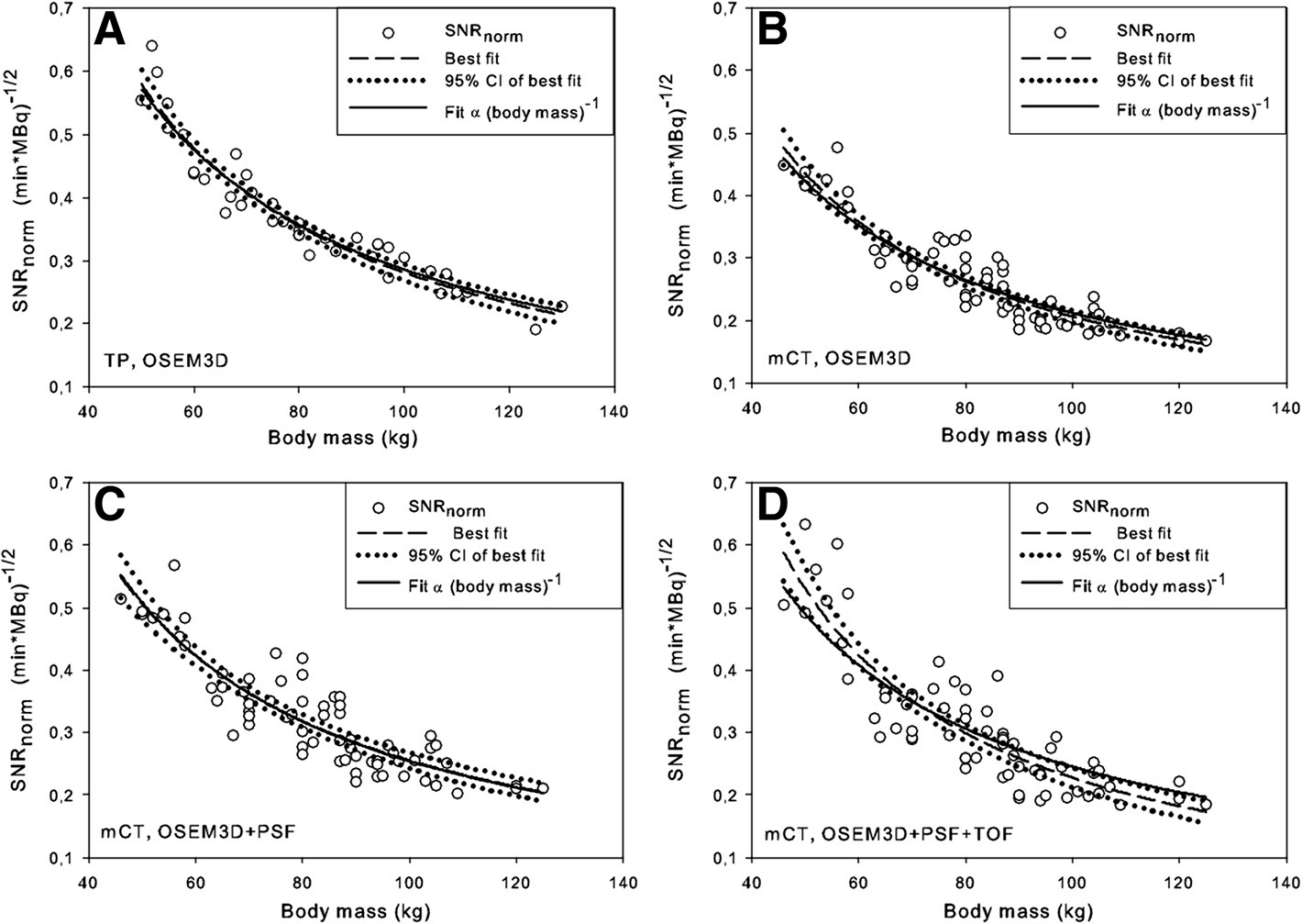
**SNR**_**norm **_**versus body mass.** Signal-to-noise ratio normalized for the administered FDG dose and scan time per bed position (SNR_norm_) versus body mass. Besides the best fits through the data, also their 95% confidence intervals are shown, and the best fit with the value of the parameter *d* fixed to 1 for **(A)** the Biograph TruePoint (TP) and for the Biograph mCT for three different reconstructions: **(B)** OSEM3D, **(C)** OSEM3D + PSF and **(D)** OSEM3D + PSF + TOF. The fit with the parameter *d* fixed to 1 corresponds to the situation where SNR_L_ can be kept constant by a quadratic relation between dose and body mass.

The results of the fits of SNR_norm_, against various patient-dependent parameters, as well as the *p* values of the *F* test compared to body mass are given in Table 3. Table 3 shows that based on the values of *R*^2^, the best fits are those where the body mass and mass per length are used as the patient-dependent parameters. We chose to use the body mass further on in the analysis because this will be the easiest to implement in the clinic and its fit with SNR_norm_ has the highest *R*^2^. In Figure 4, the fits and their 95% CI of the SNR_norm_ with the patient’s body mass are shown for both PET/CT cameras.

**Table 3:** **Fit of SNR**_**norm **_**with various patient-dependent parameters**

**Patient-dependent parameter**	**Biograph TruePoint**				**Biograph mCT**											
	**OSEM3D**				**OSEM3D + PSF + TOF**				**OSEM3D + PSF**				**OSEM3D**			
	***a***	***d***	***R***^**2**^	***p*value of *F *test**	***a***	***d***	***R***^**2**^	***p*value of *F *test**	***a***	***d***	***R***^**2**^	***p*value of *F *test**	***a***	***d***	***R***^**2**^	***p*value of *F *test**
Body mass (kg)	31.43	1.02	0.93	-	63.57	1.22	0.77	-	24.47	0.99	0.79	-	30.19	1.08	0.84	-
BMI (kg/m^2^)	11.16	1.04	0.78	<0.001*	19.20	1.28	0.52	0.003*	11.28	1.10	0.56	0.006*	13.11	1.20	0.59	<0.001*
Lean body mass (Hume, kg)	63.07	1.30	0.72	<0.001*	90.26	1.42	0.68	0.200	37.69	1.19	0.69	0.145	47.30	1.30	0.72	0.058
Fat mass (Hume, kg)	1.60	0.47	0.80	0.001*	2.01	0.60	0.64	0.065	1.61	0.51	0.67	0.081	1.56	0.56	0.71	0.035*
Lean body mass (James, kg)	20.41	1.01	0.49	<0.001*	46.46	1.24	0.65	0.119	21.44	1.03	0.65	0.086	25.68	1.13	0.69	0.030*
Fat mass (James, kg)	1.39	0.44	0.75	<0.001*	1.66	0.57	0.56	0.009*	1.37	0.49	0.59	0.010*	1.31	0.54	0.63	0.002*
Mass per length (kg/m)	23.49	1.09	0.91	0.325	49.14	1.34	0.71	0.400	24.16	1.13	0.74	0.513	29.97	1.24	0.78	0.363

Data were fitted with a first-order polynomial (Equation 4). *p < 0.05.

### Second part of the study

For the second part of the study, the SNR_acc_ was determined to be 9.58, corresponding to the image quality of a patient with a body mass of 75 kg in the linear dose regimen. According to Equation 6 and using the fit data from Table 3, this gives the optimized dose regimen (*A* in MBq, *m* in kg):

(7) $$\begin{array}{l} A=\frac{1}{t}{\left(\frac{{\mathrm{SNR}}_{\mathrm{acc}}}{a}\right)}^{2}\cdot m^{2d}\\ \;=\frac{1}{4}{\left(\frac{9.58}{31.43}\right)}^{2}\cdot m^{2.047}=0.023\cdot m^{2.047}\;\mathrm{MBq}. \end{array}$$

**Figure 5:**
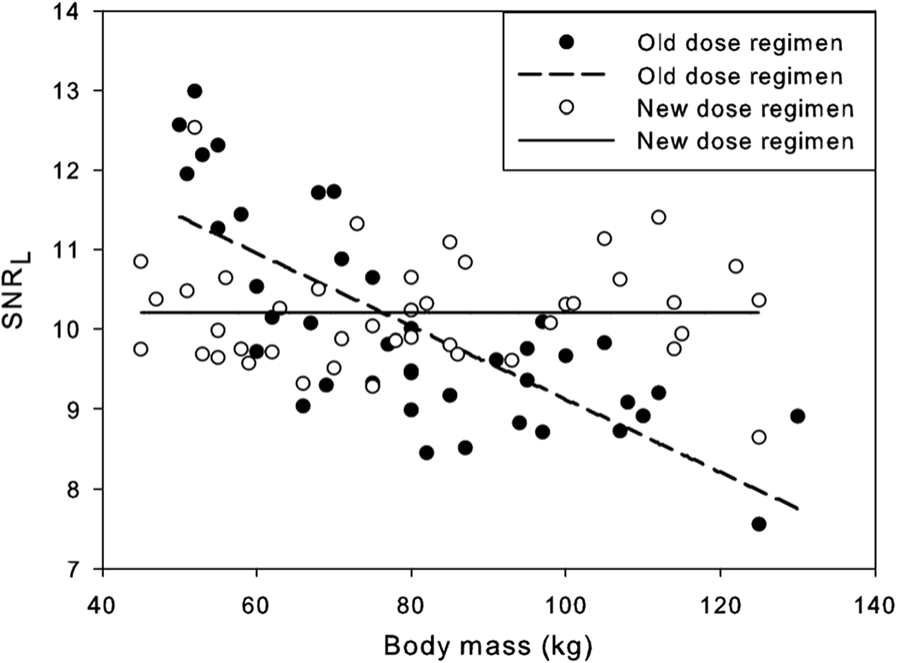
**Comparison between the old and new dose regimens.** SNR_L_ versus body mass for the old and new dose regimens for the Biograph TruePoint.

This new dose regimen for the Biograph TruePoint (Equation 7) was validated for a group of 42 new patients. The results are shown in Figure 5. It is seen from this figure that the SNR_L_ no longer decreases with body mass (*p* = 0.98). This is also illustrated by the patient scans in Figure 3C,D. The body masses of these two patients are equal to the body masses of the patients in Figure 3A,B. However, the image quality for the heavier patient is now similar to that of the lighter patient.

### Simulation study

**Figure 6:**
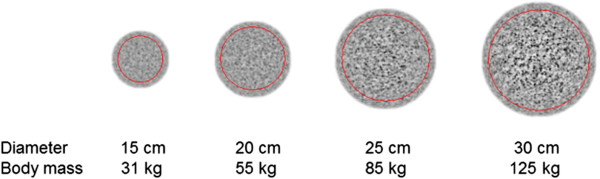
**Axial slices of the simulated mathematical phantom.** Representative axial slices of the simulated mathematical phantom with various diameters. Images were taken from the simulation applying a linear relationship between weight and administered activity. The region of interest to derive SNR is indicated by the red contour.

**Figure 7:**
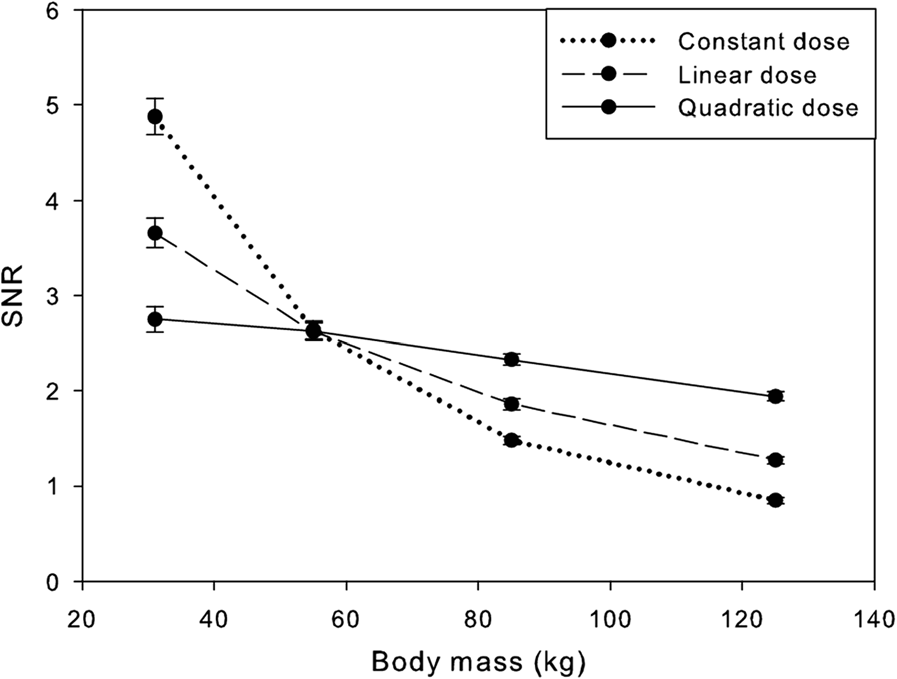
**Simulation results.** SNR versus the body mass represented by the phantom for constant, linear and quadratic dose regimes using AW-OSEM reconstruction. The error bars represent two standard deviations.

Figure 6 shows representative axial slices of the simulated mathematical phantom with various diameters. The results of the simulations were well in line with clinical observations. The use of a fixed amount of FDG dose resulted in a large change in image quality or decreasing SNR with increasing phantom diameter, i.e. increasing body mass (Figure 7). Using a linear relation between body mass and FDG dose, this reduction was still approximately a factor of 2 between phantoms representing patients having a body mass of 55 and 125 kg, respectively (Figure 7). When simulated administered FDG dose was proportional to the square of the patient’s body mass, image noise remained fairly constant (within 30% difference) across the range of simulated patient’s body masses.

## Discussion

The current EANM guidelines [1] advise a linear relation between FDG dose and body mass. The data in Figure 2C,E,G were obtained using a linear relation between the patient’s body mass and the FDG dose, but the scan time per bed position was varied for different classes of body mass. However, this adaption in the scan time can be corrected for, based on Equation 2. The results of this correction are shown in Figure 2D,F,H. From these figures, it is clearly seen that the EANM guidelines result in a decreasing SNR_L_ with increasing body mass for the Biograph mCT for all reconstructions used in this study. The regression lines in Figure 2A,B were obtained using the data of all patients scanned on the Biograph TruePoint in the first part of the study. However, patients with a body mass above 90 kg received a higher FDG dose per kilogram of body mass for every kilogram over 90 kg (see Table 1). This higher dose might compensate for the decline of SNR_L_ for heavier patients. However, if two separate regression analyses are performed for these two classes of body mass, it turns out that for both groups the SNR_L_ decreases significantly with body mass (*p* < 0.001 and *p* = 0.03, respectively). So even though patients with a body mass above 90 kg were administered a higher FDG dose per kilogram of body mass, the SNR_L_ also decreases with body mass for this group on the Biograph TruePoint. The first part of the study indicated that for both cameras a quadratic relation between FDG dose and body mass should result in a more constant SNR_L_ and that this is valid for all the reconstruction methods used. Validation of this new dose regimen showed that a quadratic dose regimen actually results in a more constant SNR_L_. These results are consistent with clinical observations, indicating that the use of the SNR as measured in the liver is a good parameter to investigate the relation between body parameters and image quality. The reason that the liver was chosen is that this is the only organ that has a relative homogeneous uptake of FDG. However, SNR_L_ also reflects variability in physiological uptake. Since heterogeneity of liver uptake was the only exclusion criterion for this study, it is expected that our results can be extrapolated to all FDG whole-body scans.

The fit of SNR_norm_ to different patient-dependent parameters shows that the fit with body mass has the highest *R*^2^, followed by mass per length (the difference of *R*^2^ is not significant for both cameras). Fits of SNR_norm_ with lean body mass, fat mass and BMI turned out to have lower values of *R*^2^, sometimes significantly so. Based on these findings and the fact that body mass is the easiest patient-dependent parameter to use, the choice for body mass was made. It may be surprising that fits of SNR_norm_ with lean body mass, fat mass and BMI have lower values of *R*^2^ than the fit with body mass as one would assume that body shape should influence image quality. Apparently, in our population, this is only a minor effect since body mass already explains between 77% and 93% of the variability in SNR_norm_. The remaining error in the fit of SNR_norm_ with body mass showed no trend with the patient’s body mass (data not shown), indicating that including additional powers of body mass will not improve the value of *R*^2^ of the fit of SNR_norm_ with body mass. Of course, the effect of other body parameters may be hidden in the remaining unexplained variance. However, a multi-variate fit would overcomplicate the application of improved dose regimes and probably reduce inter-subject variability only marginally. Since the fit of SNR_norm_ with body mass has the highest *R*^2^ and is easily obtained and a very practical parameter to use, only the relation between SNR_norm_ and body mass was considered further.

Analyzing the data, we determined that a quadratic relation between the patient’s body mass and administered dose should result in a more constant SNR_L_, i.e. an image quality that is less dependent of the patient’s body mass. From Table 3, it is seen that for both TOF and non-TOF systems, the fitting parameter *d* is approximately, but not exactly equal to, 1. The obtained relationship between the patient’s body mass and the administered FDG dose (Equation 6) is therefore not exactly quadratic. The standard deviation of the relative error distribution of the fits varies, depending on the reconstruction used, from 7.3% to 15.3%. To determine the influence of this deviation of the parameter *d* of the value of 1, the best fit between SNR_norm_ and body mass for *d* = 1 is displayed in Figure 4 too. It is seen from Figure 4 that for three out of the four cases the fit with *d* = 1, which would result in an exact quadratic relation between the patient’s body mass and the FDG dose, lies within the 95% CI of the best fit. Only for the Biograph mCT, OSEM3D + PSF + TOF reconstruction, the fit for *d* = 1 lies partly outside the 95% CI of the best fit. However, this deviation is small. Therefore, it was reasonable to state that the optimal relation between the patient’s body mass and the FDG dose is quadratic.

Equation 6 was based on the assumption that the SNR scales with the square root of the measured counts (Equation 2), i.e. that *N* is proportional to *A*, where *A* is the amount of activity at the time of administration. This assumption was checked for OSEM3D, OSEM3D + PSF and OSEM3D + PSF + TOF reconstructions by performing scans of different acquisition times of a cylindrical phantom uniformly filled with ^68^Ge (data not shown). The activity in the phantom was low enough to avoid dead time effects. The fit of the SNR with the scan time showed that SNR is proportional to *t*^*n*^, where the parameter *n* varied from 0.44 to 0.47, depending on the reconstruction used. However, it turns out that if these exact values are used in the analysis, the values of *d* do not change significantly. For both cameras, the waiting period between the administration of FDG and the start of the PET acquisition was standardized (1 h) and the total scan times were relatively short compared to the half-life of FDG, which makes it reasonable to assume that *N* is proportional to *A*. On the Biograph mCT, different scan times per bed position were used for different classes of body mass. This introduces a difference in the time between FDG administration and the start of the acquisition of the PET slice containing the liver for patients with different body masses. However, this difference will be only a couple of minutes, which is short compared to the half-life of FDG. Therefore, the use of Equation 2 was justified.

As we have shown, the product of the dose and the time per bed position determines the SNR_L_ (Equation 6). Therefore, the acquisition time per bed position can, in principle, be varied without influencing the image quality, as long as the administered FDG dose is changed accordingly. However, this study assumes that other effects which influence image quality such as dead time correction as well as random and scatter coincidences are small. Therefore, one should be careful when increasing the FDG dose in favour of a shorter acquisition time per bed position because dead time effects can influence the image quality negatively. In this study the noise equivalent count rate (NECR) remained in the linear range with respect to the administered FDG dose. However, if higher doses are applied, which is, e.g. the case in the USA [16], this is no longer the case, and dead time effects become more important. For very heavy patients and without adapting the scan time, the quadratic dose regimen results in very high levels of administered dose and system dead time should then be monitored. In addition to the decrease of dead time effects, the use of longer acquisition time per bed position for obese patients also has the benefit of reducing the radiation burden for both the patient and the technician.

A quadratic dose regimen may be considered less practical than a linear relation, although this should not be a problem when using automated dispensing units or a lookup table. Alternatively, one could also linearize the quadratic relation in parts or use a linear dose regimen while adapting the acquisition time per bed position with the patient’s body mass to mimic the quadratic relation according to Equation 6. The latter approach was used on the Biograph mCT, resulting in the data of Figure 2C,E,G.

The value of SNR_acc_ was in this study only determined for the OSEM3D reconstruction of the Biograph TruePoint camera. Because noise has a different structure in PSF and PSF + TOF reconstructions compared to OSEM3D reconstructions, the values of the SNR of different reconstruction methods are not directly comparable, and the value of SNR_acc_ has to be determined separately for different reconstruction methods. The analysis in this paper was performed on two Siemens cameras. Apparently, for the systems tested, the quadratic relation is valid for OSEM3D with or without PSF or PSF + TOF reconstructions. This does not automatically mean that the obtained relationship is also valid for cameras of different manufacturers or for different reconstruction methods. Ideally, for these cameras and reconstruction methods, one should repeat the analysis of the SNR_L_. Nevertheless, we would not be surprised if our results hold for other systems too.

Under this assumption, the new, quadratic dose *A*_*q*_ (MBq) for a patient with body mass *m* (kg) depends on the old, linear dose *A*_lin_ (MBq) for this patient through Equation 8 (see Additional file 1):

(8) $$A_{q}=\left(\frac{m}{m_{T}}\right)\cdot A_{\mathrm{lin}},$$

where *m*_*T*_ is the maximum body mass up to which the image quality is considered acceptable in the linear dose regimen.

The results of the simulations were in line with clinical observations and confirm that at FDG activities as recommended by EANM (approximately in the range of 180 of 260 MBq for a patient with a body mass of 75 kg) [1], image quality, expressed as SNR, remained fairly constant across patients with different body masses when the amount of FDG dose administered was proportional with the square of the patient’s body mass. Simulations suggest that this relationship can be primarily explained by the increased attenuation with increasing tissue mass as dead time and contribution of random coincidences and scattered photons were not included. Although the latter omissions can be considered as a limitation of the simulations, it should be noted that typical FDG activities administered in Europe [1] are much lower than those applied in, for example, the USA [16,17]. Consequently, PET/CT studies in Europe are usually operated in the linear part of the NECR curve, where the contributions of dead time and random coincidences have a much smaller effect on NECR than at higher FDG activities where the NECR curve becomes flat [4]. SNR in patients can be influenced by physiology as well. Changes in plasma clearance, obesity and/or plasma glucose levels can have an effect on the biodistribution of FDG and thus SNR. However, this study shows that these effects are either rare or not as important as the effect of attenuation. For example, by simply simulating effects of attenuation on image quality (SNR), we were able to closely replicate the clinical findings, i.e. a linear relation between the patient’s body mass and administered FDG dose was not sufficient to achieve uniform image quality across patients. By proportionally scaling the FDG dose with the square of the body mass, a more uniform image noise level as a function of (simulated) patient body mass can be achieved. For patients heavier than approximately 120 kg, the simulations indicate that an FDG dose proportional to a higher power of the body mass is needed to obtain the same SNR as for lighter patients. However, patients in this range of body mass are rare in our settings and thus rare in our analysis. Therefore, we cannot compare in full these specific simulation results to clinical data.

The lowest patient’s body mass in this study was 45 kg. It remains to be shown whether our results can be extrapolated to lower body masses. In addition, it also important to note that the results obtained in this paper are only valid for adults. For children, one should therefore use current international guidelines such as those from the EANM [18] or optimized dose regimes such as [9].

## Conclusion

Using a dose regimen based on [1], in which the relation between the administered FDG dose and the patient’s body mass is linear, a decreasing image quality for obese patients was observed. Of the tested patient-dependent parameters, fits of body mass and mass per length with image quality had the highest *R*^2^. The body mass was chosen because it is the easiest parameter to implement in the clinic. A quadratic relation between FDG dose and the patient’s body mass is recommended as both simulations and clinical observations confirm that image quality remains constant across patients when this dose regimen is used.

## Competing interests

The authors declare that they have no competing interests.

## Authors’ contributions

EHdG analyzed the data, performed the statistical analysis and drafted the manuscript. NP analyzed the data. RB designed and performed the simulations. NRLW provided the patient data. ATMW participated in the design of the study and revised the manuscript. JAvD designed the study, performed the statistical analysis and revised the manuscript. All authors read and approved the final manuscript.

## Supplementary Material

Additional file 1 **Clinical implementation of the optimized dose regimen.** A derivation is given on how to extract the recommended quadratic relation between dose and body mass, directly from the linear dose strategy that is currently applied in clinical practice. Eventually, it results in determining the maximum body mass up to which the image quality is considered acceptable in the linear dose regimen. Click here for file

## References

[B1] Boellaard R O’Doherty MJ Weber WA Mottaghy FM Lonsdale MN Stroobants SG Oyen WJG Kotzerke J Hoekstra OS Pruim J Marsden PK Tatsch K Hoekstra CJ Visser EP Arends B Verzijlbergen FJ Zijlstra JM Comans EFI Lammertsma AA Paans AM Willemsen AT Beyer T Bockisch A Schaefer-Prokop C Delbeke D Baum RP Chiti A Krause BJ FDG PET and PET/CT: EANM procedure guidelines for tumour PET imaging: version 1.0 Eur J Nucl Med Mol Imaging 2010 3 1 181 200 10.1007/s00259-009-1297-4 19915839 PMC2791475

[B2] Boellaard R Oyen WJG Hoekstra CJ Hoekstra OS Visser EP Willemsen AT Arends B Verzijlbergen FJ Zijlstra J Paans AM Comans EFI Pruim J The Netherlands protocol for standardisation and quantification of FDG whole body PET studies in multi-centre trials Eur J Nucl Med Mol Imaging 2008 3 12 2320 2333 10.1007/s00259-008-0874-2 18704407

[B3] Matsumoto K Matsuura H Minota E Sakamoto S Nakamoto Y Senda M Evaluation of optimized injection dose and acquisition time using body mass index for three-dimensional whole-body FDG-PET Nihon Hoshasen Gijutsu Gakkai Zasshi 2004 3 11 1564 1573 15568009 10.6009/jjrt.kj00003326581

[B4] Watson CC Casey ME Bendriem B Carney JP Townsend DW Eberl S Meikle S Difilippo FP Optimizing injected dose in clinical PET by accurately modeling the counting-rate response functions specific to individual patient scans J Nucl Med 2005 3 11 1825 1834 16269596

[B5] Everaert H Vanhove C Lahoutte T Muylle K Caveliers V Bossuyt A Franken PR Optimal dose of 18F-FDG required for whole-body PET using an LSO PET camera Eur J Nucl Med Mol Imaging 2003 3 12 1615 1619 10.1007/s00259-003-1317-8 14504831

[B6] Halpern BS Dahlbom M Auerbach MA Schiepers C Fueger BJ Weber WA Silverman DHS Ratib O Czernin J Optimizing imaging protocols for overweight and obese patients: a lutetium orthosilicate PET/CT study J Nucl Med 2005 3 4 603 607 15809482

[B7] Halpern BS Dahlbom M Quon A Schiepers C Waldherr C Silverman DH Ratib O Czernin J Impact of patient weight and emission scan duration on PET/CT image quality and lesion detectability J Nucl Med 2004 3 5 797 801 15136629

[B8] Masuda Y Kondo C Matsuo Y Uetani M Kusakabe K Comparison of imaging protocols for 18F-FDG PET/CT in overweight patients: optimizing scan duration versus administered dose J Nucl Med 2009 3 6 844 848 10.2967/jnumed.108.060590 19443586

[B9] Accorsi R Karp JS Surti S Improved dose regimen in pediatric PET J Nucl Med 2010 3 2 293 300 10.2967/jnumed.109.066332 20080887

[B10] Conti M State of the art and challenges of time-of-flight PET Phys Med 2009 3 1 1 11 10.1016/j.ejmp.2008.10.001 19101188

[B11] Conti M Focus on time-of-flight PET: the benefits of improved time resolution Eur J Nucl Med Mol Imaging 2011 3 6 1147 1157 10.1007/s00259-010-1711-y 21229244

[B12] El Fakhri G Surti S Trott CM Scheuermann J Karp JS Improvement in lesion detection with whole-body oncologic time-of-flight PET J Nucl Med 2011 3 3 347 353 10.2967/jnumed.110.080382 21321265 PMC3088884

[B13] Panin VY Kehren F Michel C Casey M Fully 3-D PET reconstruction with system matrix derived from point source measurements IEEE Trans Med Imaging 2006 3 7 907 921 16827491 10.1109/tmi.2006.876171

[B14] Hume R Prediction of lean body mass from height and weight J Clin Pathol 1966 3 July 389 392 5929341 10.1136/jcp.19.4.389 PMC473290

[B15] Hallynck TH Soep HH Thomis JA Boelaert J Daneels R Dettli L Should clearance be normalised to body surface or to lean body mass? Br J Clin Pharmacol 1981 3 523 526 10.1111/j.1365-2125.1981.tb01163.x 7272167 PMC1401592

[B16] Graham MM Badawi RD Wahl RL Variations in PET/CT methodology for oncologic imaging at U.S. academic medical centers: an imaging response assessment team survey J Nucl Med 2011 3 2 311 317 10.2967/jnumed.109.074104 21233185 PMC3889016

[B17] Beyer T Czernin J Freudenberg LS Variations in clinical PET/CT operations: results of an international survey of active PET/CT users J Nucl Med 2011 3 2 303 310 10.2967/jnumed.110.079624 21233186

[B18] Stauss J Franzius C Pfluger T Juergens KU Biassoni L Begent J Kluge R Amthauer H Voelker T Højgaard L Barrington S Hain S Lynch T Hahn K Guidelines for 18F-FDG PET and PET-CT imaging in paediatric oncology Eur J Nucl Med Mol Imaging 2008 3 8 1581 1588 10.1007/s00259-008-0826-x 18536914

