# Supplementary material for: Optimized dose regimen for whole-body FDG-PET imaging
Source: EJNMMI Res. 2013 Aug 12;3:63. doi: 10.1186/2191-219X-3-63 (PMC3751692; doi:10.1186/2191-219X-3-63)
Supplement: Additional file 1 — Clinical implementation of the optimized dose regimen. A derivation is given on how to extract the recommended quadratic relation between dose and body mass, directly from the linear dose strategy that is currently applied in clinical practice. Eventually, it results in determining the maximum body mass up to which the image quality is considered acceptable in the linear dose regimen. [file 2191-219X-3-63-S1.pdf]

Clinical implementation of the optimized dose regimen.

Assuming that the quadratic relation is valid for a specific setting, the new relation between FDG dose and body mass can easily be obtained following a simple procedure. The starting point is the dose regimen that is currently being used in the clinic. Assuming a fixed scan time per bed position and a linear relation between body mass (m, in kg) and FDG dose (A_{lin}, in MBq), this can be written as:

$$A_{lin} = c \cdot m \qquad (i)$$

with *c* the FDG dose per kg. The new, quadratic dose regimen can be described by:

$$A_q = h \cdot m^2 \qquad (ii)$$

with *h* the FDG dose per kg squared and A_q the FDG dose in MBq. A nuclear medicine expert has to determine up to which maximum body mass m_T the image quality is considered adequate in the old dose regimen. By requiring that for m_T the dose in the quadratic regimen is identical to the corresponding dose for the linear model A_T, equation (ii) can be solved directly to give:

$$A_q = h \cdot m^2 = \left(\frac{A_T}{m_T^2} \right) \cdot m^2 \qquad (iii)$$

Note that, using equation (i) and the fact that $A_T = c \cdot m_T$, equation (iii) can also be rewritten to exemplify the relation between the linear dose and the quadratic dose as shown in equation (iv):

$$A_q = \left(\frac{A_T}{m_T^2} \right) \cdot m^2 = \left(\frac{m}{m_T} \right) \cdot A_{lin} \qquad (iv)$$

Which equals equation (8) in the paper.
In the new dose regimen, a patient with a body mass equal to the threshold body mass m_T will receive the same FDG dose as in the old dose regimen. Patients with a body mass higher than m_T will receive a higher dose, while patients with a body mass lower than m_T will receive less FDG than in the old dose regimen.
